# Understanding how post-intensive care follow-up is delivered within the role of critical care outreach teams: a qualitative study protocol

**DOI:** 10.1136/bmjopen-2026-117840

**Published:** 2026-05-24

**Authors:** Amy Bonner, Owen Gustafson, Sophie Betteridge, Natalie Pattison, Peter J Watkinson, Sarah Vollam

**Affiliations:** 1 Critical Care Research Group, Nuffield Department of Clinical Neurosciences, University of Oxford, Oxford, UK; 2 Nursing, Midwifery and Allied Health Professions Research and Innovation, Oxford University Hospitals NHS Foundation Trust, Oxford, UK; 3 School of Health, Medicine and Life Sciences, University of Hertfordshire, Hatfield, UK; 4 East and North Hertfordshire NHS Trust, Stevenage, UK; 5 Oxford Critical Care, Oxford University Hospitals NHS Foundation Trust, Oxford, UK

**Keywords:** Clinical Protocols, QUALITATIVE RESEARCH, Adult intensive & critical care

## Abstract

**Introduction:**

Each year over 130 000 patients in the UK are discharged from an intensive care unit (ICU), with many experiencing poor outcomes such as in-hospital mortality, emergency ICU readmission and/or significant morbidity. Despite current national guidance and the availability of follow-up services, post-ICU care remains variable. Critical Care Outreach Teams (CCOTs) are key in supporting this patient group, yet practice differs considerably. Recovery pathways have been successfully employed in other patient populations and are a potential option to standardise post-ICU care. Understanding how care is currently delivered by CCOT throughout the UK is essential to inform future development of an evidence-based recovery pathway for this patient group. Our primary aim is to understand how post-ICU follow-up care is delivered within the wider remit of CCOT workloads.

**Methods and analysis:**

This is a pragmatic multicentre qualitative study of post-ICU follow-up care. The study will be split into two sub-studies: semi-structured interviews and ethnographic observations. Semi-structured interviews will be conducted with three groups of individuals: multi-professional staff members involved in the care of patients discharged from ICU to the wards, patients discharged from ICU to the wards and their family members. Direct participant observations alongside ad hoc informal discussions will be undertaken with members of the CCOT at participating sites focusing on their workflow to generate an understanding of the CCOT role and how post-ICU support fits within this. An overarching thematic analysis approach will be taken to analyse data from both sub-studies to clearly identify the barriers and facilitators to providing post-ICU support within the CCOT role.

**Ethics and dissemination:**

Ethical approval has been obtained through the London—Chelsea Research Ethics Committee (25/PR/0773). We aim to disseminate the findings to local teams, at regional and international conferences, in peer-reviewed journals and through social media.

**Trial registration number:**

ISRCTN14138257.

**STRENGTHS AND LIMITATIONS OF THIS STUDY**

- This study will integrate findings from both semi-structured interviews and ethnographic observations to provide a nuanced understanding of how post-intensive care unit follow-up care is delivered.
- Data will be collected from five diverse UK centres with varying service delivery models, geographical locations and patient populations which were chosen in collaboration with patient and public involvement partners.
- Ethnographic observations will be undertaken at different timepoints within the clinical working day to capture fluctuations in activity.
- There is a potential for participants to alter their behaviour and practice during ethnographic observations which could introduce bias.

## Introduction

 Over 130 000 patients are discharged from non-specialist intensive care units (ICUs) in the UK each year.^1^ Of these, almost 10 000 either die on the ward or are unexpectedly readmitted to an ICU. In addition to these poor outcomes, there is significant morbidity associated with recovery from critical illness.^2^,^5^ Despite National Institute for Health and Care Excellence (NICE) guidelines for post-ICU recovery and rehabilitation being available since 2009,^6^ against which services are commissioned, problems in care are evident throughout the acute hospital admission which contribute to these poor outcomes.^7^ Patients and their family members also report discharge from intensive care as a frightening experience where they commonly feel unsupported.^8^ These challenges have been further highlighted by the recent UK National Confidential Enquiry into Patient Outcome and Death (NCEPOD) report, which identified significant shortfalls in rehabilitation provision in hospital following critical illness.^9^

These problems in care and poor outcomes occur despite three quarters of ICUs providing an in-hospital follow-up/recovery service to support care after ICU discharge.^10 11^ Post-ICU support provision in UK hospitals varies widely. Critical Care Outreach Teams (CCOT), available in 85% of the UK, are recommended to visit patients at least once after ICU discharge and within the first 24 hours of transfer, but there is no ongoing support recommendation and practice varies.^12^ At-risk patients may be seen for longer, but this is discretionary.^13^ Parent medical teams have ultimate responsibility for this care provision. As part of wider Rehabilitation After Critical Illness (RACI) or ICU follow-up/recovery, some services include ward-based follow-up as usual care.^10 14^ In this protocol, for brevity, CCOT will be used as an overarching term to encompass all healthcare professionals within teams (CCOT, RACI and/or in-hospital ICU follow-up or recovery services) who aim to support the ward team with all patients (including those discharged from ICU), help identify and support patients who are deteriorating and facilitate communication between settings.^14^,^16^ They commonly follow local protocols or national guidance based on limited evidence^10 14^ with ICU follow-up visits often ceasing 24–48 hours after transfer to the ward, despite ongoing clinical problems.^7^

Care provision has been successfully standardised in other patient populations (eg, stroke and major surgery) through the use of recovery pathways.^17 18^ However, unlike in other conditions, post-ICU patients are not discharged from ICU to a single location, but are distributed throughout the hospital based on the specialty of their original reason for ICU admission.^19^ Given this geographical spread and the differing underlying skills of staff in the receiving specialty wards, any future care pathway for critical care survivors will need to be supported by CCOT.

The National Institute of Health and Care Research (NIHR) funded Enhanced Recovery After Critical Care (ERACC) programme of work (NIHR206266) aims to develop and test an evidence-based, theoretically driven, electronically enabled, in-hospital enhanced recovery pathway for patients following discharge from ICU. In order to develop a pathway that could be supported by CCOT, it is essential to understand how current UK CCOT practices are delivered, and how it sits within the local, regional and national health requirements. This includes an in-depth understanding of the breadth of provision, structure of CCOTs within hospital cultures, and their interactions.

## Methods and analysis

### Aim and objectives

The aim is to understand how post-ICU follow-up care is delivered within the wider remit of CCOT and/or in-hospital ICU follow-up workloads.

Specifically, the study intends to understand:

1. The different models of post-ICU care delivery.
2. What proportion of CCOT time is spent on following ICU patients post-ICU discharge and how this is prioritised (eg, internal/external drivers).
3. The competing demands on CCOT time.
4. The experiences of CCOT staff of providing ICU follow-up care within their role.
5. The wider staff perceptions of current ICU follow-up provision and perception of what post-ICU in-hospital care should look like.
6. Patient and family member perceptions of post-ICU in-hospital follow-up provision.

### Patient and public involvement

Our patient and public involvement (PPI) representatives who are co-applicants of the wider ERACC programme of work were actively consulted throughout the development of this study. They have had input into the study design and have been involved in selecting the sites for the study (to ensure representativeness of the population), guiding the approach to participants, and developing the patient-facing documents and the interview topic guides.

### Study design

This is a pragmatic multicentre qualitative study of post-ICU follow-up care. Data collection is split into two sub-studies: semi-structured interviews and ethnographic observations. Semi-structured interviews will be held with patients discharged from ICU, their family members, and staff providing care to post-ICU patients. Members of the CCOT and/or post-ICU follow-up services, where available, at selected sites will be observed undertaking their role with ad-hoc informal interviews taking place with participants to deepen understanding of observed interactions.

### Study settings

This qualitative study will be undertaken across five separate UK NHS Trusts, selected for their variation in geographical location, size and clinical provision across their CCOT and post-ICU in-hospital follow-up services.

### Participant selection

#### Semi-structured interviews

Any clinical staff member involved in the care of patients discharged from ICU at the five sites will be eligible to participate. This includes members of CCOT (some of whom had specific remit for RACI/ post-ICU follow-up or recovery services), multi-professional ward staff such as nurses, doctors, clinical support workers, physiotherapists and dieticians. Invitations to participate will be disseminated by e-mail by the local research lead or Principal Investigator (PI), advertised by posters displayed in the clinical areas, and staff members may also be approached by researchers during observations. Expressions of interest to participate will be sent to the research team via e-mail. Staff participants will be purposively selected to achieve maximum variation in the sample, aiming to capture a broad range of clinical experience and professions.^20^ CCOT members from across the UK may also express interest in this study during participation in a national survey (Open Science Framework doi: 10.17605/OSF.IO/B5XJ3) conducted as part of the wider ERACC programme of research.

Patients over the age of 18 years discharged from ICU to the ward, and their family member will be invited to participate during their post-ICU ward stay. Patient and family member participants will be purposefully sampled to include a wide range of experiences. We will aim to include patients who have had varying durations of critical illness and ongoing care needs, which we will also aim to mirror in the family member interviews. Patients discharged from ICU at the five sites and their family members will be given information about the study by CCOT members at each site, during their routine visits. ICU discharge lists may also be screened by local research delivery teams who may approach patients about the study. Potential family members will be identified by patients during patient approach.

#### Ethnographic observations

All members of the CCOT (including RACI and ICU follow-up practitioners) at the five sites will be eligible to participate. Invitations to participate will be disseminated as above, with expressions of interest sent to the research team. We plan to include a maximum variation sample of team members in terms of clinical experience, grade and profession within the CCOT and follow-up teams.

### Consent

#### Semi-structured interviews

Staff who contact the research team to express interest in participating will be informed of the aims of the study and given a participant information sheet. If after considering the information provided the staff member is willing to participate, contact will be made to arrange a suitable date and venue for the interview. Written informed consent will be gained prior to the interview. If a participant has been identified from the national survey, consent will be taken remotely using a remote staff participant consent.

Patients who have made contact with the research team (either directly or through the CCOT) will be approached by the researchers and asked if family members may also be willing to participate. Consultee consent will not be sought from the patient’s next of kin. Potential participants will be informed of the aims of the study and given a participant information sheet. Written informed consent will be obtained prior to the interview.

#### Ethnographic observations

Consent will be managed using both ‘opt-in’ and ‘opt-out’ approaches. For CCOT members at each site who contact the research team to express interest in participating, the approach outlined above for seeking informed consent for staff interviews will be followed. These individuals will consent to being directly observed in clinical practice.

An opt-out option will be available to all professionals whose interactions with the CCOT members are observed. An opt-out form can be completed by anyone wishing to opt out of observations and returned to the research team via email or internal post to their local ICU lead for the study. A list of those who have opted out and their place of work will be kept and cross-checked prior to and during each observation. Posters will be displayed in clinical areas outlining the study and informing staff of their opt-out options.

### Sample size

#### Semi-structured interviews

We will interview up to 30 staff, patient and family member participants. This is deemed to be sufficient to provide a detailed account of post-ICU support from a multitude of perspectives based on previous work and the concept of information power,^20^ with the study having a relatively focused research question but aiming to include a wide variety of participant perspectives from different staff groups and patient experiences.

#### Ethnographic observations

We will complete up to 10 periods of observation each lasting up to 4 hours at each participating site to provide a maximum of 200 hours. This is deemed sufficient based on our focused research question and relatively limited variety in participants.^19^

### Data collection

#### Semi-structured interviews

We will conduct semi-structured interviews with three groups of individuals: multi-professional staff members involved in the care of patients discharged from ICU to the wards, patients discharged from ICU to the wards and family members. This will allow us to explore and contrast a range of individuals’ perspectives of ongoing care delivered following the transition from ICU to the wards.

The process for interviewing healthcare professionals, patients and family members will be similar. Staff interviews will be held in person in a private room in the hospital away from the clinical area, or by video calling (using MS Teams) or telephone, depending on participant preference. Patients and family members may be interviewed together (as dyadic interviews), or separately, depending on participant preference. Interviews will be conducted either in hospital, if the patient is well enough and willing to do so (as deemed by the clinical team and person themselves), or at home after hospital discharge. In-hospital interviews will be conducted on the ward in a private room or at the patient bedside if sufficiently private. Post-hospital interviews will be held by telephone or video call within 3 months of hospital discharge, allowing patients time to recover from their critical illness but retaining sufficient proximity to the event to allow recall. If the interview is taking place after hospital discharge, a member of the research team will take contact details (phone number, email address) to arrange a mutually convenient time.

Interviews are expected to last between 30 and 60 min, will follow a topic guide (online supplemental material) and will be audio recorded. The topic guide was based on previous work,^21^ established literature and input from patient and public representatives. It included open-ended questions and prompts to explore the experiences and perception of ICU follow-up care provision.^22^ The topic guide was piloted on expert clinical staff and adjusted based on feedback. It is anticipated that the topic guide will evolve throughout the interviews to ensure any generated themes are explored,^23^ reflecting the iterative nature of qualitative research.

At the start of the interview, the interviewer will confirm consent to participate, explain that the session will be audio recorded and that they may stop the interview at any time. Interviews will last around 30–60 min and be recorded using a separate audio recording device (OLYMPUS Digital Voice Recorder VN-541PC). Interviews will follow a topic guide including questions and prompts focused around support provided by CCOTs to patients discharged from ICU. Audio recordings will be transcribed verbatim, either by the research team or using the QSR NVIVO transcription function (https://lumivero.com/products/nvivo-transcription/).

#### Ethnographic observations

Direct participant observations will be undertaken with consented CCOT members. Each observation period will last for up to 4 hours, with the period of observation varying throughout the day (and potentially overnight), to capture variations in workflow across shifts. Up to ten periods of observation will be undertaken at each site, with participants potentially being observed on multiple occasions. A member of the research team will shadow the consented CCOT member(s)—this may include more than one team member—during the observation period. Observation will focus on workflow of CCOT, including but not restricted to supporting patients discharged from ICU.

Although observations will be focused on the workflow of the consented CCOT member(s), we will observe CCOT members interacting with other staff members, including other members of the CCOT and wider multiprofessional team members (such as ward nurses, doctors or allied health professionals, ICU doctors and nurses and operations managers). Shadowing participants and observing interactions with other staff members will allow for a more complete understanding of the CCOT role and how post-ICU support fits within this. Non-participant observations will be undertaken (with the observer playing no part in interactions).

Direct care delivery will not be observed to protect patient privacy. Instead, informal discussions will be undertaken with CCOT members following visits to patients to capture brief details about the types of interventions provided during patient contact (eg, advice provided, vital signs measured, mouth care provided, etc) and to understand clinical prioritisation.^19^ These ad-hoc informal discussions will also assist to give context to the interaction and develop further understanding of the CCOT role and decision-making processes.^19^ Data (field notes, informal conversation summaries and interview notes) will be collected with an electronic or handwritten case report form, which will be piloted prior to first use.

CCOT members participating in this phase may also participate in the staff interviews.

### Data analysis

Interviews and field notes will be transcribed verbatim into a specialist software package for coding qualitative data (QSR NVIVO, https://lumivero.com/products/nvivo/). An overarching thematic analysis approach will be taken to analyse these qualitative data, based on Braun and Clark’s six steps.^24^ This will ensure clear identification of the barriers and facilitators to providing post-ICU support within the CCOT role, and suits the pragmatic aims of this study. This approach has previously been used to identify areas of care which patients and staff believed could be improved.^25 26^

Data from each sub-study will initially be analysed separately, using the same approach. Qualitative data from interview transcriptions, ethnographic participant observation field notes and informal discussions will be analysed using an inductive–iterative approach, aided by reflexive notes. To support reflexivity, reflexive diaries and regular supervision with an experienced ethnographer will aid data collection and analysis.^19^

Preliminary coding will take place soon after the interviews/observations are conducted, with each data set analysed separately initially, and then together. This will allow any identified themes to be explored in subsequent data collection. Preliminary coding will be refined as outlined by Braun and Clark to identify preliminary themes.^24^ Analysis will continue until preliminary themes are refined into the final theme structure. Throughout preliminary coding, codes and developing themes will be discussed within the research team at regular meetings. Once initial analysis of each data set is complete, data will be further analysed using cross-case comparison, across each case and type of data, using inductive analysis techniques based on thematic analysis.^19 27^ Themes will be compared across data sets to identify commonalities, differences, and where the two approaches can contribute to build a more comprehensive picture of post-ICU support.^28^

Once finalised, a report will be produced, reporting the most important themes across the two data sets, and representing the full range of experiences included in the interviews (both research and conversational ethnographic interviews) and observations. For the final output, these themes will be further categorised by aspects of the system which could be improved and suggestions for improvement.

Credibility will be achieved through triangulation of data from the two methods (interviews and ethnography), including identification of similarities and differences from the two approaches. Confirmability will be ensured through careful field notes and reflexive accounts during data collection (collected as part of the case report form), regular meetings of the research group throughout analysis, to discuss codes and developing themes, and research diaries of decisions made in data collection and analysis to provide an audit trail. Transferability will be maximised by seeking sites with differing post-ICU service provision, patient populations, and hospital size and setting, as well as including participants from outside these sites identified through the external UK survey.

## Ethics and dissemination

### Ethics

The study received ethical approval from the London—Chelsea Research Ethics Committee 25/PR/0773. The University of Oxford will act as sponsor. The study, as part of the wider ERACC programme, will be overseen by a programme steering committee and includes PPI involvement throughout.

This manuscript reports Version 1 of the study protocol (June 2025) and has been written in relation to Standard Protocol Items: Recommendations for Interventional Trials (SPIRIT)^29^ and consolidated criteria for reporting qualitative research (COREQ) checklist^30^.

Interviews have the potential to cover some distressing topics (such as the pressure of responding to emergencies and providing good care in a pressurised environment for staff and the potential to recall poor care delivery for patients). To protect participants, we have a protocol in place for managing potential distress during interviews with patients and family members. Staff, patient and family members will be free to stop a scheduled interview or the interview itself at any point.

Ethnographic observations have the potential to be stressful or intrusive for staff, particularly when responding to emergency situations. Researchers will be given written support information and signposted to occupational health in their NHS Trust in the first instance (Distress Protocol available at https://doi.org/10.1186/ISRCTN14138257).

We will only seek consent from the CCOT members being directly observed as it is not possible to seek consent from every professional they may interact with. However, observations are focused on CCOT workload and interactions will only be observed in the context of how they influence CCOT workflow, therefore minimal data will be collected related to other staff. No direct patient care interactions will be observed.

### Dissemination

Findings from this study will be disseminated to local teams, at regional and international conferences, in peer-reviewed journals and through social media. Authorship of any papers related to this study will follow the ICMJE recommendations (http://www. icmje.org/ recommendations/).

## Supplementary material

10.1136/bmjopen-2026-117840 online supplemental file 1
