# Supplementary material for: Understanding how post-intensive care follow-up is delivered within the role of critical care outreach teams: a qualitative study protocol
Source: BMJ Open. 2026 May 24;16(5):e117840. doi: 10.1136/bmjopen-2026-117840 (PMC13202168; doi:10.1136/bmjopen-2026-117840)
Supplement: online supplemental file 1 [file bmjopen-16-5-s001.docx]

**ERACC Semi-Structured Interview Example Topic Guide 1 – Staff Members**

| **Core Themes to Explore** | The current role of outreach/follow-up services in supporting patients |
| --- | --- |
|  | How this fits in with the wider remit of CCOT |
|  | What could be improved or changed |
| **Example questions** | How are critical care outreach/follow-up services provided in your clinical setting? |
|  | Could you tell me about your experiences of looking after patients on the ward following intensive care discharge, particularly thinking about how outreach/follow-up services support this? |
|  | What do outreach/follow-up staff do to support post-ICU patients on the ward? |
|  | Do you have any concerns about this process? |
|  | How does supporting post-ICU patients fit within the wider workload of outreach teams? |
|  | Would you make any changes to the service to better support patients discharged from ICU to the ward? |
|  | What happens when patients become unwell after discharge from ICU? |
|  | Are there any other factors you think are relevant to the management of patients transferred from intensive care? |
|  | Who should I talk to, to find out more? |

**ERACC Semi-Structured Interview Example Topic Guide 2 – Patient and Family Members**

| **Core Themes to Explore** | The current role of outreach/follow-up services in supporting patients |
| --- | --- |
|  | How patient and family members perceive this support |
|  | What could be improved or changed |
| **Example questions** | Did you receive any support from ICU staff when you were moved to the ward? If so, can you tell me about this? |
|  | Could this support have been improved? |
|  | Did you have enough support from ICU teams? |
|  | Do you have any concerns about this process? |
|  | Would you make any changes to the service to better support patients discharged from ICU to the ward? |
|  | Did you ever become unwell after discharge from ICU? If so, did ICU teams come and see you then? |
|  | Are there any other factors you think are relevant to the management of patients transferred from intensive care? |
|  | Who should I talk to, to find out more? |
