# Supplementary material for: Understanding how post-intensive care follow-up is delivered within the role of critical care outreach teams: a qualitative study protocol
Source: BMJ Open. 2026 May 24;16(5):e117840. doi: 10.1136/bmjopen-2026-117840 (PMC13202168; doi:10.1136/bmjopen-2026-117840)
Supplement: Supplementary file 2 [file bmjopen-2026-117840.reviewer_comments.pdf]

PEER REVIEW HISTORY

BMJ Open publishes all reviews undertaken for accepted manuscripts. Reviewers are asked to complete a checklist review form and are provided with free text boxes to elaborate on their assessment. These free text comments are reproduced below.

ARTICLE DETAILS

Title (Provisional)

Understanding how post-intensive care follow-up is delivered within the role of Critical Care Outreach Teams: a qualitative study protocol

Authors

Bonner, Amy; Gustafson, Owen; Betteridge, Sophie; Pattison, Natalie; Watkinson, Peter J; Vollam, Sarah

---

This article was not externally reviewed at BMJ Open. Protocols that have been independently assessed prior to submission to BMJ Open are usually fast-tracked to publication on the grounds that further substantial changes will not be possible. This independent assessment will usually be external, independent review for both a substantial grant award from a non-commercial or government funder and ethics approval.
